# Non-destructive and efficient method for obtaining miRNA information in cells by artificial extracellular vesicles

**DOI:** 10.1038/s41598-023-48995-5

**Published:** 2023-12-14

**Authors:** Fumio Maeda, Shungo Adachi, Tohru Natsume

**Affiliations:** 1 Cellular and Molecular Biotechnology Research Institute, National Institute of Advanced Industrial Science and Technology, 2-3-26 Aoumi, Koto-ku, Tokyo, 135-0064 Japan; 2 Department of Proteomics, National Cancer Center Research Institute, 5-1-1 Tsukiji, Chuo-ku, Tokyo, 104-0045 Japan

**Keywords:** Diagnostic markers, Biological techniques, Biotechnology, Biomarkers

## Abstract

In recent years, research has explored the use of microRNA (miRNA) analysis in extracellular vesicles (EVs) as a minimally invasive strategy for the diagnosis and prediction of diseases. This is because miRNAs in EVs partly reflect the miRNA information and cellular status of the origin cells. However, not all intracellular miRNAs are internalized into EVs. Therefore, the miRNA information obtained from EVs is limited. To get more miRNA information, we aimed to produce artificial EVs (aEVs) encapsulating Argonaute 2 (Ago2) miRNA-binding protein, which actively incorporate miRNAs within themselves. In this study, we utilized the protein EPN-01, which is capable of releasing aEVs encapsulating it and associated proteins. This system enables us to obtain more miRNA species and increase each miRNA’s yield in the EV fraction. Furthermore, we examined whether miRNAs in the EV fraction using our system reflect the cellular condition. In cells treated with CoCl_2_, a reagent for inducing a hypoxia-mimic state, we detected a change in the level of hypoxia marker miR-210 with aEVs. To the best of our knowledge, this is the first report on a method to increase the yield and variety of endogenous miRNAs in the EV fraction. This approach leads to improved accuracy of cell status assessment using miRNAs in EVs.

## Introduction

Methods to evaluate cell status through cell cultures or body fluids enable the identification of cell conditions in a non-destructive manner. These methods are a subject of intense research for their potential application to the diagnosis of diseases^1–3^. Extracellular vesicles (EVs) are secreted from cells and carry biomolecules, such as microRNAs (miRNAs), that are present in the origin cells^4,5^. Emerging evidence has shown these miRNAs reflect the state of the cells and can be used as biomarkers^6,7^.

miRNAs are short non-coding RNAs that are approximately 22 nucleotides in length that regulate gene expression through targeting mRNAs. After export from the nucleus to the cytoplasm, miRNAs are loaded into Argonaute (Ago) proteins^8^. The miRNAs direct Ago proteins to a target site in the 3′ untranslated region of mRNAs, and the miRNA-Ago complex induces mRNA cleavage or inhibition of translation, resulting in gene silencing^8^. Several miRNAs are encapsulated within EVs through the packaging of their associated RNA-binding proteins^9^. Recent reports have suggested that these miRNAs enclosed in EVs are employed for cell-to-cell communication^10^. miRNA expression profiles differ depending on the cell type and developmental stage of cells, and studies have demonstrated that various diseases, such as neurodegenerative disorders and cancer, are associated with characteristic miRNA expression profiles^11–14^. The profiles of miRNAs encapsulated in EVs also vary depending on cell conditions or disease states. For example, the miRNA profiles in EVs purified from the blood, plasma, and urine of patients with lung cancer, prostate cancer or liver cancer are different compared with those obtained from healthy patients^15–18^. Owing to the characteristics of EV miRNAs in bodily fluids, examining them is expected to enable disease diagnosis in a less invasive manner than the performance of invasive biopsies and surveying cells. However, only a small fraction of intracellular miRNAs is encapsulated in EVs and, in normal unengineered cells, extracellular miRNA is rarely abundantly expressed and is less dynamic under different pathophysiological conditions^19,20^. Therefore, we hypothesized that the additional production of EVs containing more biomarker miRNA species and high levels of miRNAs would improve the sensitivity of miRNA detection compared with evaluating basal levels of miRNAs in endogenous EVs.

Enveloped protein nanocage (EPN)-01 is an artificially designed protein that induces the formation of EVs^21,22^. EPN-01 is composed of four domains: (i) an N-myristylation signal corresponding to the first six amino acids of human immunodeficiency virus type 1 (HIV-1) structural Gag protein for membrane binding, (ii) I3-01, which forms icosahedral nanocages by self-association, (iii) a Myc tag, and (iv) the p6 domain of HIV-1 Gag, which recruits endosomal sorting complexes required for transport (ESCRT) proteins and interacts with the HIV protein Vpr^21,23^. A previous study showed that when EPN-01, which contains a domain that interacts with Vpr-fused proteins, and the vesicular stomatitis viral glycoprotein (VSV-G), which is a membrane fusion protein, were co-expressed in cells, EPN-01 nanocages encapsulated the Vpr-fused protein and the EPN-01 nanocages induced the budding of EVs containing EPN-01 nanocages from the plasma membrane^21^. The EVs then fuse with target cells to deliver their contents^21^. While the previous study reported the use of EV induction by EPN-01 for the purpose of protein delivery between cells, the EVs could be used for obtaining intracellular material by fusing Vpr to a protein that binds to the material. We hypothesized that by producing EVs encapsulating a Vpr-fused miRNA-binding protein such as Ago protein by the EPN-01 nanocages, it would be possible to efficiently obtain miRNAs from the EV fraction.

In this study, we co-expressed an Ago protein fragment fused with Vpr and EPN-01 to induce EVs containing the Ago fragment with miRNAs. Co-expression of the Ago fragment and EPN-01 increased the variety of miRNA species and yield of each miRNA detected in the EV fraction. Finally, to examine whether changes in cellular conditions could be detected by investigating EV fractions of miRNA, we subjected the cells to hypoxic conditions and determined whether changes in the hypoxic marker miR-210 could be detected^24,25^. In a hypoxic state, the expression of miR-210 is upregulated; moreover, miR-210 has regulatory functions on a variety of cellular processes, which endow it with the potential to affect the etiology of various cancers and non-cancerous disorders^24,25^. When cells were treated with CoCl_2_, a reagent for inducing a hypoxia-mimicking state^26^, co-expression of EPN-01 and the Ago fragment enabled detection of the increase in miR-210 in the EV fraction with high sensitivity.

## Results

### MID-PIWI domain of Ago2 was detected in the extracellular vesicle fraction with EPN-01 in HEK293T cells and bound with miRNA let7a-5p

**Figure 1:**
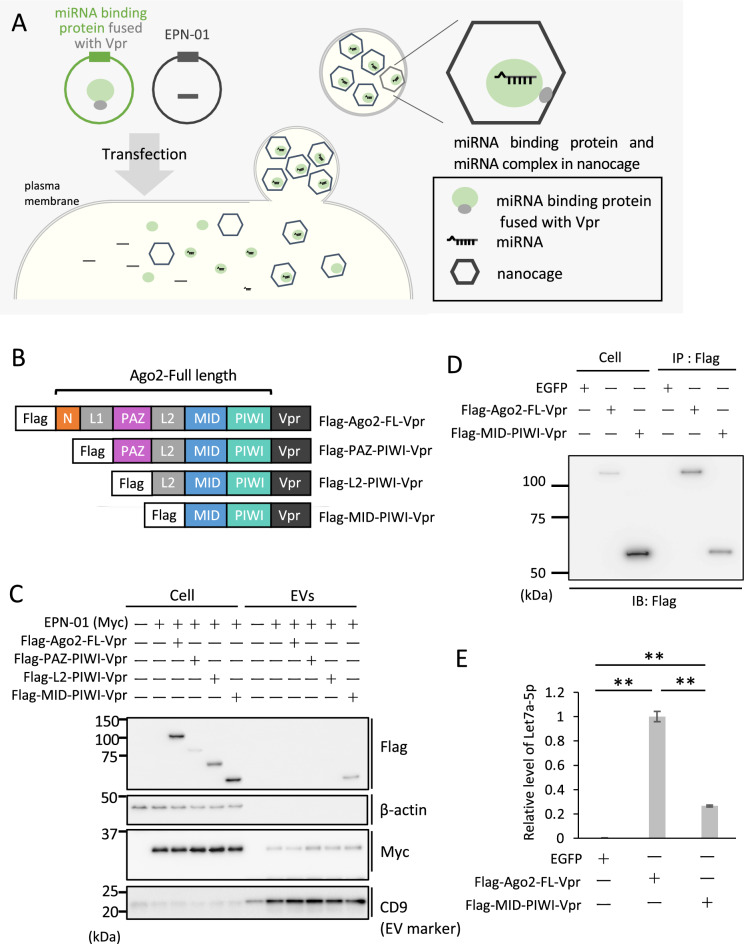
Schematic overview of the production of miRNA-containing extracellular vesicles. (**A**) EPN-01 nanocages containing miRNA-binding protein fused with Vpr and miRNAs budding at the plasma membrane. (**B**) Schematic of Ago2 truncation mutants fused with Flag-tag and Vpr. (**C**) Immunoblot analysis showing Ago2-FL and the truncated mutants fused with Vpr and Flag-tag and EPN-01 (Myc tag) harvested from transfected HEK293T cells (Cell) or the EV fraction of the cell culture supernatants (EVs). (**D**) HEK293T cells transfected with EGFP, Flag-Ago2-Vpr or Flag-MID-PIWI-Vpr plasmids were immunoprecipitated (IP) and analyzed by immunoblot analysis. (**E**) Relative level of let7a-5p co-immunoprecipitated with Ago2 or its truncated mutant MID-PIWI fused with Flag-tag and Vpr. Data shown were calculated using the comparative CT (ΔΔCT) method. After normalization to the input, the data were expressed relative to the mean of the control, which was then normalized to 1; results are representative of three independent experiments. Error bars represent the standard deviation. Statistical analysis was performed by one-way ANOVA and Tukey’s test. **p < 0.01.

To produce miRNA-rich EVs (Fig. 1A), we used Ago2, which binds to the majority of miRNAs compared with Ago1 or Ago3^27^. Ago2 has four distinctive domains [amino (N) terminal domain, Piwi-Argonaut-Zwille (PAZ) domain, MID domain, P-element induced wimpy testes (PIWI) domain] and two linker domains (L1, L2) (Fig. 1B)^8^. The PAZ domain interacts with the 3′ end of the miRNA, the MID domain interacts with the 5′ end of the miRNA, and the PIWI domain holds the 5′ end of the miRNA^8,28^. The L2 domain is required for the formation of a microRNA-induced silencing complex (miRISC) with several proteins including TNRC6A/GW182^8,29^. As Ago2 is thought to form complexes with miRNAs, mRNAs, and proteins in the cell, it might not be encapsulated into EPN-01 nanocages, which are approximately 20 nm in diameter^8,28,29^. We prepared plasmid vectors expressing full-length Ago2 (Ago2-FL) or truncated mutants fused with a Flag-epitope tag (Flag-tag) at the N terminal end and Vpr at the C terminal end (Fig. 1B) and examined which fragment of Ago2 was internalized into EVs. These plasmids were co-expressed with plasmid expressing EPN-01 in HEK293T cells and the intracellular proteins and EV fraction purified from conditioned medium were analyzed by immunoblot. In line with the previous study^21^, EPN-01 was detected in the EV fraction (Fig. 1C). Only Flag-MID-PIWI-Vpr was observed in the EV fraction, while full-length Ago2 or the truncated mutants were not detected (Fig. 1C).

**Figure 2:**
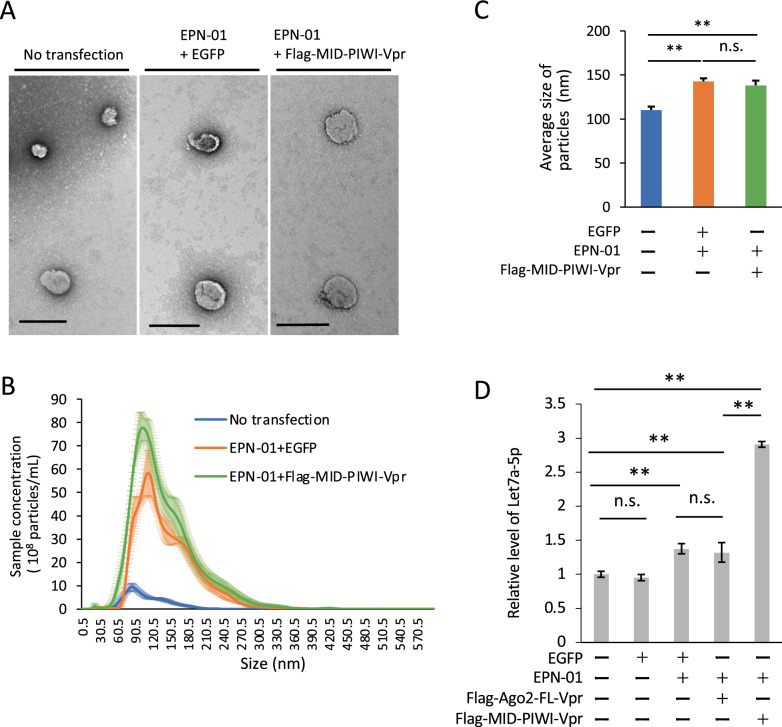
EPN-01 and MID-PIWI-Vpr increase the number of extracellular vesicles and the yield of let7a-5p in HEK293T cells. (**A**) HEK293T cells were left untransfected (control) or transfected with EGFP and Flag-MID-PIWI-Vpr. Representative electron microscopy negative staining images of EVs of the EV fraction. Scale bar 200 nm. (**B**) Size distribution profiles of particles in the EV fractions as determined by nanoparticle tracking analysis (NTA). Data are the mean ± standard error of three independent experiments. (**C**) The average size of particles was measured by NTA. Data are mean ± standard error of three independent experiments. (**D**) Relative level of let7a-5p from the EV fraction of non-transfected (control) or transfected HEK293T cells. PCR reactions for each sample were carried out in triplicate. Data shown are 2^CT^ values expressed relative to the mean determined for control, which was normalized to 1; results are representative of three independent experiments. Error bars represent the standard deviation. Statistical analysis was performed by one-way ANOVA and Tukey’s test. **p < 0.01.

To examine whether Flag-MID-PIWI-Vpr binds to miRNA, we transfected plasmid expressing the enhanced green fluorescence protein (EGFP), Flag-Ago2-FL-Vpr or Flag-MID-PIWI-Vpr in HEK293T cells and performed immunoprecipitation using anti-Flag antibody (Fig. 1D). Total RNAs were purified from the precipitate and miRNA let7a-5p, which is relatively abundant in HEK293T cells (miRmine, https://guanfiles.dcmb.med.umich.edu/mirmine/index.html), was evaluated by RT-qPCR. The amount of let7a-5p that co-precipitated with Flag-MID-PIWI-Vpr was decreased compared with the amount that co-precipitated with Flag-Ago2-FL-Vpr, but significantly (p < 0.01) higher compared with that of the negative control EGFP (Fig. 1D and E). The above results indicate that the MID-PIWI domain of Ago2 is a candidate miRNA-binding protein for encapsulation into EVs formed by EPN-01.

### EPN-01 and Flag-MID-PIWI-Vpr increased the number of EVs and the yield of let7a-5p in HEK293T cells

Next, we examined the number and size of particles in the extracellular fraction of transfected cells purified by sucrose cushion ultracentrifugation. Electron microscopy showed purified vesicles in the EV fraction (Fig. 2A). Nanoparticle tracking analysis showed that the expression of EPN-01 increased the number and size of particles in the EV fraction (Fig. 2B and C). We further examined the yield of miRNAs in the EV fraction. Plasmids were transfected into HEK293T cells, and protein accumulation in cells and EV fractions was confirmed by immunoblot (Fig. S1). As shown in Fig. S1, the expression levels of CD9 (EV marker) and EPN-01 (Myc) within the cells and the yield in EV fractions were similar among cells co-transfected with EPN-01. This suggested that there is no significant change in the yield of EVs among these cells. Flag-MID-PIWI-Vpr was also detected in the EV fraction, but Flag-Ago2-FL-Vpr was not (Fig. S1). Furthermore, co-expression of EPN-01 with EGFP or Flag-Ago2-FL-Vpr slightly increased the yield of let7a-5p in the EV fraction compared with controls, but without a statistically significant difference between EGFP and Flag-Ago2-FL-Vpr (Fig. 2D). It is speculated that the increase in yield of let7a-5p in the EV fraction is due to the altered EV production rate. As expected, co-expression of EPN-01 and Flag-MID-PIWI-Vpr significantly increased the yield of let7a-5p (p < 0.01) in the EV fraction compared with the other transfections (Fig. 2D). These results suggest that the co-expression of EPN-01 and Flag-MID-PIWI-Vpr produces EVs encapsulating Flag-MID-PIWI-Vpr, which incorporates let7a-5p into EVs.

### Co-expression of EPN-01 and Flag-MID-PIWI-Vpr increased both the variety of miRNA species and the yield of miRNAs in extracellular vesicle fractions

**Figure 3:**
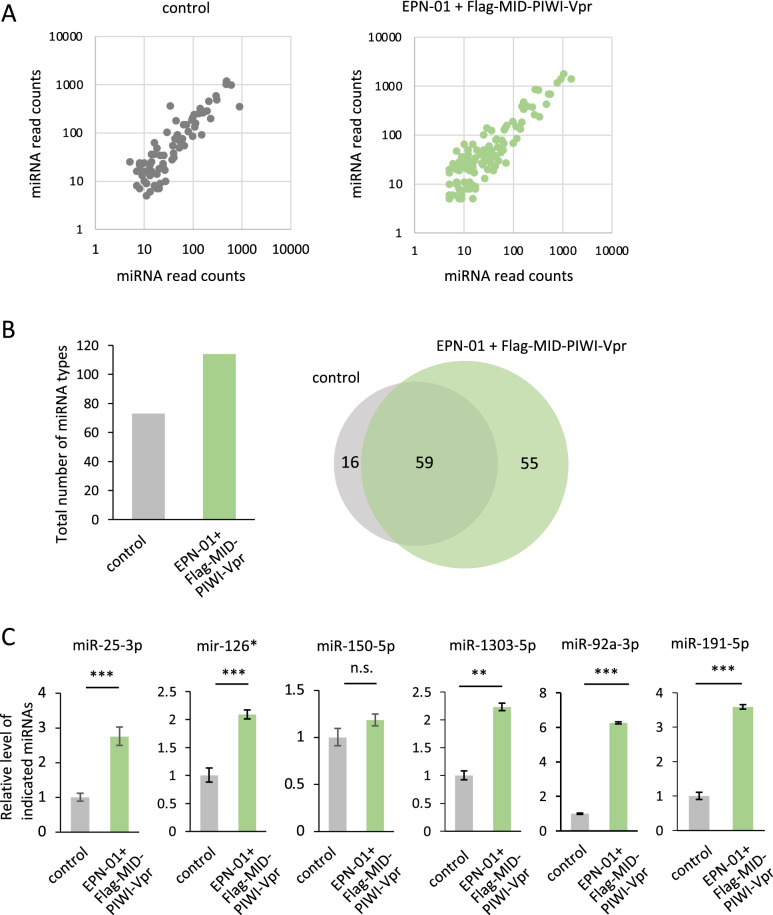
EPN-01 and MID-PIWI-Vpr transfection increases both the variety of miRNA species and the level of miRNAs from transfected HEK293T cells. (**A**) The read counts for the detected miRNAs (with a threshold of ≥ 5 read counts) obtained from two independent experiments were calculated and plotted on the x- and y-axes. (**B**) (left) Total number of miRNA species in the fraction of non-transfected (control) or transfected (EPN-01 + Flg-MID-PIWI-Vpr) HEK293T cells. (right) Venn diagrams of miRNAs in the EV fraction of non-transfected (control) or transfected (EPN-01 + Flag-MID-PIWI-Vpr) HEK293T cells. We analyzed miRNAs with a read count of 5 or more in two independent experiments. (**C**) RT-qPCR showing the relative levels of indicated miRNAs from non-transfected (control) or transfected (EPN-01 + Flag-MID-PIWI-Vpr) HEK293T cells. PCR reactions for each sample were carried out in triplicate. Data shown are 2^−ΔCT^ value expressed relative to the mean determined for the control, which was normalized to 1; results are representative of three independent experiments. Error bars represent the standard deviation. **p < 0.01, ***p < 0.001 according to Student’s *t*-test. Student’s *t*-test was performed using the Ct values of three independent experiments.

To investigate whether co-expression of EPN-01 and Flag-MID-PIWI-Vpr increases the variety of miRNA species detected in the EV fraction, we examined miRNAs in the EV fraction using next-generation small RNA-seq. The top 10 miRNA species obtained from each fraction in the two experiments are listed in Table 1. A total of 75 miRNA species were identified in the EV fraction of control cells and 114 miRNA species were identified in that of cells co-transfected with EPN-01 and Flag-MID-PIWI-Vpr, with 59 overlapping miRNA species and 16 and 55 miRNA species detected in only control cells and cells co-expressing EPN-01 and Flag-MID-PIWI-Vpr, respectively (Fig. 3A and B). As shown in Fig. S2, compared to the control, the correlation score (R square) between miRNAs obtained from cells and miRNAs from EV fractions was higher when EPN-01 and Flag-MID-PIWI-Vpr were transfected.

**Table 1:** Top 10 miRNAs obtained in experiments 1 and 2 from the extracellular vesicle fractions of control cells or cells co-transfected with EPN-01 and Flag-MID-PIWI-Vpr constructs using next-generation small RNA-seq.

1st experiment			
Control		EPN-01 + Flag-MID-PIWI-Vpr	
miRNA	Read counts	miRNA	Read counts
hsa-miR-25-3p	1192	hsa-miR-25-3p	1789
hsa-let-7a-5p	1020	hsa-miR-92a-3p	1421
hsa-miR-126-5p	985	hsa-miR-1303	1413
hsa-miR-150-5p	583	hsa-miR-3960	1171
hsa-miR-92a-3p	484	hsa-miR-19b-3p	855
hsa-let-7f-5p	456	hsa-miR-19a-3p	830
hsa-let-7c-5p	365	hsa-miR-4739	693
hsa-miR-1303	350	hsa-let-7a-5p	690
hsa-miR-191-5p	323	hsa-miR-6873-3p	479
hsa-miR-23a-3p	295	hsa-miR-126-5p	429

2nd experiment			
Control		EPN-01 + Flag-MID-PIWI-Vpr	
miRNA	Read counts	miRNA	Read counts
hsa-miR-1303	883	hsa-miR-1303	1483
hsa-miR-126-5p	599	hsa-miR-25-3p	1039
hsa-miR-25-3p	481	hsa-miR-92a-3p	906
hsa-let-7a-5p	468	hsa-miR-3960	774
hsa-miR-92a-3p	302	hsa-let-7a-5p	555
hsa-miR-150-5p	290	hsa-miR-4739	512
hsa-miR-19b-3p	226	hsa-miR-126-5p	464
hsa-let-7f-5p	208	hsa-miR-4488	337
hsa-miR-19a-3p	190	hsa-miR-19a-3p	319
hsa-miR-223-3p	159	hsa-miR-19b-3p	276

RT-qPCR was then performed to validate the small RNA-seq results. We evaluated the yield of six miRNA species (miR-25, miR-126*, miR-150-5p, miR-1303, miR-92a-3p and miR-191-5p), which were the six most abundant miRNAs in the EV fraction of control cells in the first RNA-seq (Table 1). The RT-qPCR results showed that co-expression of EPN-01 and Flag-MID-PIWI-Vpr increased the yield of miR-25, miR-126*, miR-1303, miR-92a-3p and miR-191-5p, but not miR-150 (Fig. 3C). Furthermore, we confirmed that the co-expression of EPN-01 and Flag-MID-PIWI-Vpr increased the yield of these miRNAs with HeLa cells (Fig. S3A and B). As with HEK293T cells, EPN-01 and Flag-MID-PIWI-Vpr were detected in the EV fraction (Fig. S3A). The RT-qPCR results showed that co-expression of EPN-01 and Flag-MID-PIWI-Vpr increased the yield of miR-25, miR-1303, miR-92a-3p and miR-191-5p, but not miR-126* and miR-150-5p (Fig. S3A). These results indicate that co-expression of EPN-01 and Flag-MID-PIWI-Vpr increases the production of EVs encapsulating miRNAs, resulting in the increase of the variety and yield of miRNAs in the EV fraction, although not all miRNAs showed an increase in yield by the co-expression of EPN-01 and Flag-MID-PIWI-Vpr.

### Co-expression of EPN-01 and MID-PIWI-Vpr enabled the detection of a hypoxia marker in the extracellular vesicle fraction

**Figure 4:**
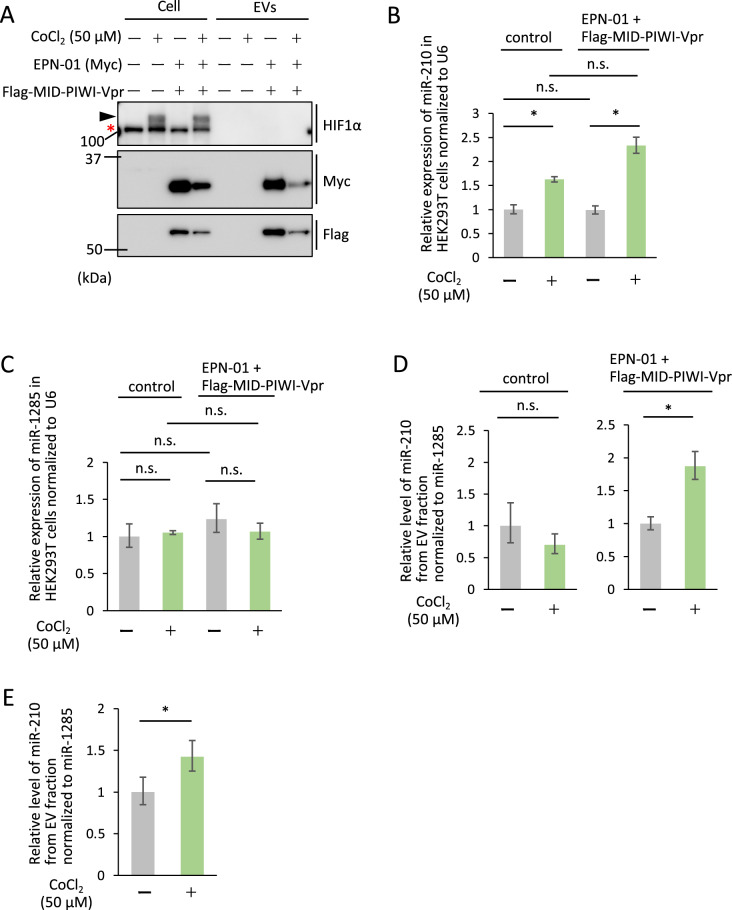
The method using EPN-01 and MID-PIWI-Vpr shows high sensitivity in detection of hypoxic cell state. (**A**) Immunoblot showing the accumulation of HIF1α, Flag-MID-PIWI-vpr, EPN-01(Myc) and β-actin in HEK293T cells or cell culture supernatants treated with CoCl_2_ (50 µM). The black arrowhead indicates HIF1α (asterisk, non-specific bands). (**B**,**C**) RT-qPCR showing the relative expression of miR-210 (**B**) or miR-1285 (**C**) in transfected or non-transfected cells treated with CoCl_2_ (50 µM). PCR reactions for each sample were carried out in triplicate. Relative expression of each miRNA was calculated using a comparative CT (ΔΔCT) method. Data were normalized to U6 small RNA and are representative of three independent experiments. (**D**) RT-qPCR showing the relative levels of miR-210 in the EV fraction of non-transfected or transfected HEK293T cell culture supernatants treated with CoCl_2_ (50 µM). PCR reactions for each sample were carried out in triplicate. Relative expression of each miRNA was calculated using a comparative CT (ΔΔCT) method. Data were normalized to miR-1285 and are representative of three independent experiments. Error bars represent the standard deviation. Statistical analysis was performed by one-way ANOVA and Tukey’s test or Student’s *t*-test. *p < 0.05, **p < 0.01.

Finally, to examine whether miRNAs in the EV fraction using our system reflect the cellular condition, we focused on hypoxia, which is induced in various conditions including bacterial infection, inflammation, wounding, cardiovascular disorders, and cancer^30^. In response to hypoxia, hypoxia-inducible factor 1α (HIF1α) is stabilized from proteasomal degradation, translocates into the nucleus, and binds to hypoxia-responsive elements, leading to upregulation of target genes including miR-210^24,30,31^. HEK293T cells were transfected with EPN-01 and Flag-MID-PIWI-Vpr, and 6 h after transfection, cells were treated for 18 h with CoCl_2,_ a reagent that causes a hypoxia-mimic state^26^. Previous studies have shown that a high concentration of CoCl_2_ in the culture medium results in accumulated cellular HIF1α, inhibits protein synthesis, and decreases the viability of the cells^26^. We used CoCl_2_ at a concentration of 50 µM, which was shown to not impact cell viability^32^. In line with previous studies^33,34^, we confirmed the accumulation of HIF-1α and miR-210 with CoCl_2_ treatment (Figs. 4A,B, S4A and B), although CoCl_2_ affected slightly the amount of protein in the cells (Figs. 4A,B, S4C and D). We used miR-1285 as a negative control miRNA; miR-1285 was the gene with the highest read counts detected in the small RNA-seq among the unreported miRNAs related to the hypoxia. No significant change was detected in the expression of miR-1285 by CoCl_2_ treatment (Fig. 4C). We investigated the yield of miR-210 in the EV fraction and normalized levels by the yield of miR-1285. CoCl_2_ treatment significantly increased miR-210 (p < 0.05) in the EV fraction of cells co-expressing EPN-01 and Flag-MID-PIWI-Vpr (Fig. 4D). In contrast, in experiments using the EV fraction from non-transfected cells, despite conducting repeated experiments, we were unable to detect a significant increase in miR-210 induced by CoCl_2_ (Fig. 4D). We attributed this to the low quantity of miRNA present in the EV fraction, which we speculated was causing issues with Realtime PCR analysis. Therefore, we performed a reanalysis using the cell culture volume sixfold, and finally, we were able to observe an increase in miR-210 expression triggered by CoCl_2_ (Fig. 4E). These results indicate that the co-expression of EPN-01 and Flag-MID-PIWI-Vpr increased the sensitivity of miRNA detection in the EV fraction.

## Discussion

In this study, we validated a system using the EV-inducing protein EPN-01 and miRNA-binding protein Ago2 as a means of obtaining miRNAs in a non-invasive and efficient manner^35^. First, we co-expressed Vpr-fused Ago2 or its truncated mutants with EPN-01 and found that the MID-PIWI domain of Ago2, lacking amino acids 1 through 454, was present in the EV fraction. Furthermore, co-expression of Flag-MID-PIWI-Vpr with EPN-01 increased the variety of miRNA species and yield of each miRNA in the EV fraction. As shown in Fig. 1C, when cells were co-transfected with Ago2 or its truncated mutants with EPN-01, the expression levels of EPN-01 within the cells and the yield in the EVs fraction were similar. However, the full-length Ago2 or its truncated mutants except for Flag-MID-PIWI-Vpr was not observed in the EVs fraction. The cause of the low expression levels of Flag-PAZ-PIWI-Vpr remains unexplained, but as Flag-Ago2-FL-Vpr and Flag-L2-PIWI-Vpr exhibited sufficient expression, it is speculated that factors other than expression levels contribute to the differences in the yield of Ago mutants in the EVs fraction. We speculate that the lack of full-length Ago2 and other mutants (without MID-PIWI) in the EVs fraction may be because the size of Ago2 complexed with proteins and/or mRNAs exceeds the size of EPN-01 nanocages, which are approximately 20 nm in diameter^21,23^. The nanocage size is defined by skeletal proteins (e.g., I3-01)^21^, and the use of skeletal proteins that make larger nanocages may contain the full-length Ago2 and other mutants. The immunoprecipitation assay indicated that the MID-PIWI domain bound miRNAs (Fig. 1E). This result agrees with a previous study in which *Drosophila* Ago1 or Ago2 mutants with deletion of the N domain to PAZ domain still bound miRNAs in cells^36^. In addition, the full-length Ago2 bound more miRNAs than the MID-PIWI domain (Fig. 1E), and therefore the substitution of I3-01 for other skeletal proteins that form larger cages than I3-01 would enable encapsulation of full-length Ago2 into these cages, resulting in an increased yield of miRNAs.

We also detected miR-210, a marker of hypoxia^24^, in cells induced to hypoxia conditions using CoCl_2_ at a low concentration with limited effects on cell viability. No increase in miR-210 was detected in the EV fraction in control cells, while a significant increase in miR-210 was observed in the EV fraction in cells co-transfected with Flag-MID-PIWI-Vpr and EPN-01. This increase may be a result of an increase in the number of EVs that took up intracellular miRNAs. These results suggest that co-expression of EPN-01 and Flag-MID-PIWI-Vpr produces miRNA-encapsulated EVs, enabling more sensitive analysis of intracellular miRNA information compared with endogenous EVs. Although it is desirable to establish appropriate quality controls when analyzing miRNA within EVs for each specific application, we speculate that this system will be applicable to detect other biomarkers.

In this study, we developed a system that promoted the production of EVs containing a high number of endogenous miRNAs. In addition, we demonstrated the utility of the system to detect the cell status by assessing hypoxia-related markers. In current blood-based miRNA analyses, one often obtains a mixture of miRNAs from various tissues, making it challenging to identify the source organ^37^. If we can safely introduce aEVs in a tissue-specific manner, it could potentially allow the acquisition of information on the particular tissue in question. Our results can be considered to constitute a preliminary step in this direction. While further studies are needed for in vivo application, we believe that this system will lead to a new approach for accurate and reliable assessment of cell status and diagnosis of disease in a non-destructive manner.

## Materials and methods

### Cells

HEK293T cells were acquired from ATCC (Rockville, MD, USA) and maintained in Dulbecco’s modified Eagle’s medium (DMEM, Wako, Osaka, Japan) containing 10% fetal bovine serum (FBS, Biosera, Nuaille, France).

### Antibodies

The following antibodies were used for western blot analysis: mouse monoclonal antibodies against Flag-tag (F-3165, Sigma-Aldrich, MO, USA), Myc (9E10, Roche, Basel, Switzerland), HIF-1α (ab51608, abcam, Cambridge, UK), rabbit polyclonal antibody against β-actin (13E5) (#4970, Cell Signaling Technology, MA, USA) and CD9 (EPR23105-121, ab236630, abcam). For immunoprecipitation, anti-Flag mouse monoclonal antibody (F1804, Sigma-Aldrich) was used.

### Plasmids

Primers used in the study were purchased from Life Technologies (Brea, Carlsbad, CA, USA) and are listed in Table 2. The plasmid vectors encoding EPN-01 and Myc-EGFP-Vpr were constructed by VectorBuilder (vector ID is VB200705-1156knk, vectorbuilder.com). Inverse PCR was performed using VB200705-1156knk as a template and primers 1 and 2 (Table 2). The resulting amplified product was digested with EcoRI and ligated to obtain pRP-Myc-EGFP-Vpr. Using the plasmid pcDNA3.2-5′F-Ago2, which encodes full-length human Ago2 cloned from human cDNA library into pcDNA3.2/V5-DEST (Invitrogen, Waltham, MA, USA) with a Flag-tag fused to the N-terminus end, as a template, PCR was performed using primers 3 and 4 (Table 2). The amplified product was inserted into the XbaI site of pRP-Myc-EGFP-Vpr using the In-Fusion^®^ HD Cloning Kit (Takara Bio USA, CA, USA) to obtain the plasmid pRP-Flag-Ago2-Vpr, which contains the sequence encoding Flag-Ago2-FL-Vpr. Using pRP-Flag-Ago2-Vpr as a template, PCR was performed using primers 5 and 6 (Table 2). The amplified product was inserted into the NheI/HindIII site of pEGFP-C2 (Takara Bio USA) by In-Fusion^®^ HD Cloning to obtain pCMV-Flag-Ago2-Vpr. PCR was performed using pcDNA3.2–5′F-Ago2 as a template and the primers 7 and 10, 8 and 10, or 9 and 10 (Table 2). The amplified product was inserted into the NheI/XbaI site of pCMV-flag-Ago2-FL-Vpr by In-Fusion^®^ HD Cloning to produce pCMV-Flag-PAZ-PIWI-Vpr, pCMV-Flag-L2-PIWI-Vpr, and pCMV-Flag-MID-PIWI-Vpr.

**Table 2:** Primer list.

No	Sequence (5′–3′)
1	GCGAATTCTTAATTAATCTAGACATGGA
2	GCGAATTCAGCCTGCTTTTTTGTACAAA
3	TTAATTAATCTAGACATGGATTACAAAGACGATGACGATAAGATGTACTCGGGAGCCGGCCC
4	ATCCTCGAGTCTAGAAGCAAAGTACATGGTGCGCA
5	GTCAGATCCGCTAGCGCCACCATGGATTACAAAGACGATGA
6	CAGAATTCGAAGCTTTTTGTACAAGAAAGCTGGGT
7	GTCAGATCCGCTAGCGCCACCATGGATTACAAAGACGATGACGATGCACAGCCAGTAATCGAGTT
8	GTCAGATCCGCTAGCGCCACCATGGATTACAAAGACGATGACGATGCAGGACAAAGATGTATTAA
9	GTCAGATCCGCTAGCGCCACCATGGATTACAAAGACGATGACGATAAGATCGAGATCAAGGTGTGGGC
10	TCCGATATCCTCGAGAGCAAAGTACATGGTGCGCA
11	GCGGTACCACCCAGCTTTCTTGTACAAA
12	GCGGTACCTTACTGAGAAGACGGGTCGTTAC
13	GTCAGATCCGCTAGCGCCACCATGGGTGCGCGTGC

To obtain the expression vector for EPN-01, inverse PCR was performed using VB200705-1156knk as a template and the primers 11 and 12 (Table 2). The amplified product was cleaved at the KpnI site and ligated to obtain pRP-EPN-01. pRP-EPN-01 was used as a template for PCR using primer 6 and 13 (Table 2). The amplified product was inserted into the NheI/HindIII site of pEGFP-C2 by In-Fusion^®^ HD Cloning to obtain pCMV-EPN-01.

### Isolation of extracellular vesicle fraction

The day before transfection, HEK 293 T cells were seeded at a density of 10^7^ cells/dish in 10 cm^2^ dishes. pCMV-EPN-01 (10 µg) was co-transfected with pEGFP-C2, pCMV-Flag-Ago2-Vpr, pCMV-Flag-PAZ-PIWI-Vpr, pCMV-Flag-L2-PIWI-Vpr, or pCMV-Flag-MID-PIWI-Vpr (5 µg) using Lipofectamine 2000 (Life Technologies). HeLa cells were seeded at a density of 2.5 × 10^6^ cells/dish in 10 cm^2^ dishes. pCMV-EPN-01 (7.5 µg) was co-transfected with pCMV-Flag-MID-PIWI-Vpr (7.5 µg) using Lipofectamine 2000 (Life Technologies). Transfection medium was replaced with 10 ml of DMEM with 10% FBS 6 h post-transfection. The culture medium was collected 24 h post-transfection, and cells and cellular debris were removed through sequential centrifugation steps at 200×*g* for 5 min and 2000×*g* for 5 min. The supernatants were centrifuged at 10,000×*g* for 30 min. The supernatants with a volume of 9.5 mL were ultracentrifuged through 2 ml of a 20% sucrose cushion at 100,000×*g* for 90 min in a swinging bucket centrifuge (Optima XL-100K, SW41 Ti rotor, Beckman Coulter, Brea, CA, USA). After a Phosphate Buffered Saline (PBS, Wako) wash, the pellet was lysed in 100 µl of Sodium Dodecyl Sulfate (SDS) sample buffer (62.5 mM Tris–HCl [pH 6.8], 20% glycerol, 2% SDS, 5% 2-mercaptoethanol) or suspended with 100 μL of PBS and collected.

### Total RNA extraction and RT-qPCR

The day before transfection, HEK 293 T cells were seeded at a density of 10^7^ cells/dish in 10 cm^2^ dishes. Transfection was performed as described above. The transfected cells were washed with PBS and collected. Total RNA was extracted from the cells or the EV lysates using the mirVana™ miRNA Isolation Kit (Invitrogen) following the manufacturer’s protocol. Total RNA was reversed transcribed using the TaqMan MicroRNA Transcription Kit (Applied Biosystems, Waltham, MA, USA) and miRNA assay (Life Technologies). miRNA-specific hairpin RT primers following the manufacturer’s instructions (miRNA assay IDs are listed in Table 3). miRNAs were amplified using TaqMan Universal Master Mix 2, no UNG (Life Technologies). miRmine (https://guanfiles.dcmb.med.umich.edu/mirmine/index.html) was used to reference miRNA abundance in HEK293T.

**Table 3:** miRNA assay ID list.

miRNA assay ID	Target
000377	hsa-let7a-5p
000451	has-miR-126-5p
000431	has-miR-92a-3p
002299	has-miR-191-5p
002792	has-miR-1303-5p
000403	has-miR-25-3p
000473	has-miR-150-5p
000512	has-miR-210-3p
002822	has-miR-1285-5p

### Immunoprecipitation

The day before transfection, HEK 293 T cells were seeded at a density of 10^7^ cells/dish in 10 cm^2^ dishes. Cells were transfected with 10 µg pEGFP-C2, pCMV-Flag-Ago2-FL-Vpr or pCMV-Flag-MID-PIWI-Vpr using Lipofectamine 2000 (Invitrogen). Transfection medium was replaced with 10 ml of DMEM containing 10% FBS 6 h post-transfection. Cells were washed with PBS 24 h post-transfection and lysed with lysis buffer (20 mM Hepes [pH 7.5], 150 mM NaCl, 50 mM NaF, 1 mM Na_3_VO_4_, 1% digitonin, 1 mM phenylmethylsulfonyl fluoride, 5 µg/mL leupeptin, 5 µg/mL aprotinin, 3 µg/mL pepstatin A). After centrifugation, the supernatants were reacted with Dynabeads™ Protein G (Invitrogen) pre-treated with anti-Flag antibody (F1804, Sigma-Aldrich) for 1 h at 4 °C with rotation. After three washes, 120 μl of Flag-elution buffer (0.5 mg/ml Flag peptide) was added and thoroughly mixed with the beads by mildly tapping the tube on ice for 5 min. The tube was placed on a magnetic stand and 100 μL of supernatant was transferred to a new collection tube. For western blotting, the supernatant was mixed with 100 µl of 2× SDS sample buffer.

### Immunoblotting

The day before transfection, HEK 293 T cells were seeded at a density of 10^7^ cells/dish in 10 cm^2^ dishes. Transfection was performed as described above. The transfected or mock-transfected cells were washed with PBS and lysed with SDS sample buffer. Cell lysates and EV lysates were subjected to electrophoresis in denaturing gels and transferred to polyvinylidene difluoride membranes. The membranes were blocked with 5% skim milk in Tris-buffered saline with Tween 20 for 30 min and reacted with indicated antibodies for at least 2 h at room temperature or 4 °C. The membranes were then reacted with secondary antibodies conjugated with peroxidase (GE Healthcare Bio-Sciences, IL, USA) for at least 1 h at room temperature. Bands were visualized using ECL (ImmunoStar LD, Wako) with LAS3000-mini (FUJIFILM, Japan). The densitograms were measured with Image J.

### Coomassie Brilliant Blue staining

Cell lysates were subjected to electrophoresis in denaturing gels and stained with SimplyBlue™ SafeStain (Life Technologies) following the manufacturer’s protocol. Protein bands were quantified with Image J.

### Small RNA sequencing and data analysis

Small RNA libraries were constructed from total RNA purified with the mirVana™ miRNA Isolation kit as described above using a SMARTer smRNA-Seq Kit (Takara Bio USA) following the manufacturer’s protocols. The cDNA library quality was checked using a 2100 Bioanalyzer (Agilent Technologies, Santa Clara, CA, USA). All libraries were prepared at 5 nM and were sequenced on an Illumina HiSeq™ 2500 platform (Illumina, Inc., CA, USA). After trimming of adapters, reads were mapped on miRBase using bowtie software. Each miRNA read count were obtained by cufflinks.

### Nanoparticle tracking analysis (NTA)

NTA was performed with a NS300 (NanoSight, Malvern, Worcestershire, UK). Camera level was set at 16 for all recordings. Samples were diluted in PBS between 1:100 and 1:1000 to achieve a particle count of between 1 × 10^8^ and 1 × 10^9^ per ml. Camera focus was adjusted to make the particles appear as sharp individual dots. Five 60-s videos were recorded for each sample. All post-acquisition functions were set at automatic, except detection threshold, which was set at 8.

### Transmission electron microscopy

Approximately 5 μl of sample was placed on a Parafilm. A carbon coated 400 mesh copper grid was positioned on the top of the drop for 10 s and washed by a droplet of distilled water. The grid was contrasted by adding a drop of 2% uranyl acetate on the Parafilm and incubating the grid on top of drop for 10 s; excess liquid was removed by gently using an absorbing paper. After drying, the sample was subjected to TEM observation using an H-7600 (HITACHI, Tokyo, Japan) at 100 kV.

### Statistical analysis

For comparisons of two groups, statistical analysis was performed using unpaired Student’s t-test. Tukey’s test was used for comparisons among multiple groups. A p-value > 0.05 was considered not significant (n.s.). All statistical analyses were performed using GraphPad Prism 7 (GraphPad Software, San Diego, CA, USA). No methods were used to determine whether the data met the assumptions of the statistical approach.

### Supplementary Information

Supplementary Figures.

## Data Availability

The datasets generated and/or analyzed during the current study are available in the Gene Expression Omnibus repository, https://www.ncbi.nlm.nih.gov/geo/query/acc.cgi?acc=GSE212033. The token is as follows: gtibeuaqrbmljyh.
