# Supplementary material for: Non-destructive and efficient method for obtaining miRNA information in cells by artificial extracellular vesicles
Source: Sci Rep. 2023 Dec 14;13:22231. doi: 10.1038/s41598-023-48995-5 (PMC10721859; doi:10.1038/s41598-023-48995-5)
Supplement: Supplementary file 1 — Supplementary Figures. [file 41598_2023_48995_MOESM1_ESM.pdf]

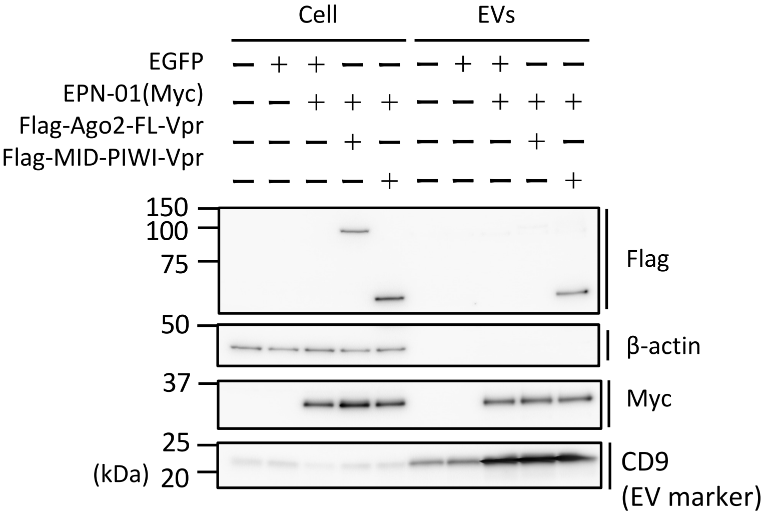

Supplemental Figure 1 Immunoblot showing Ago2 or its truncated mutants MID-PIWI fused with Flag-tag and Vpr, and EPN-01 (Myc-tag) harvested from non-transfected or transfected HEK293T cells (Cell) or the EV fraction of the cell culture supernatants (EVs).

First experiment

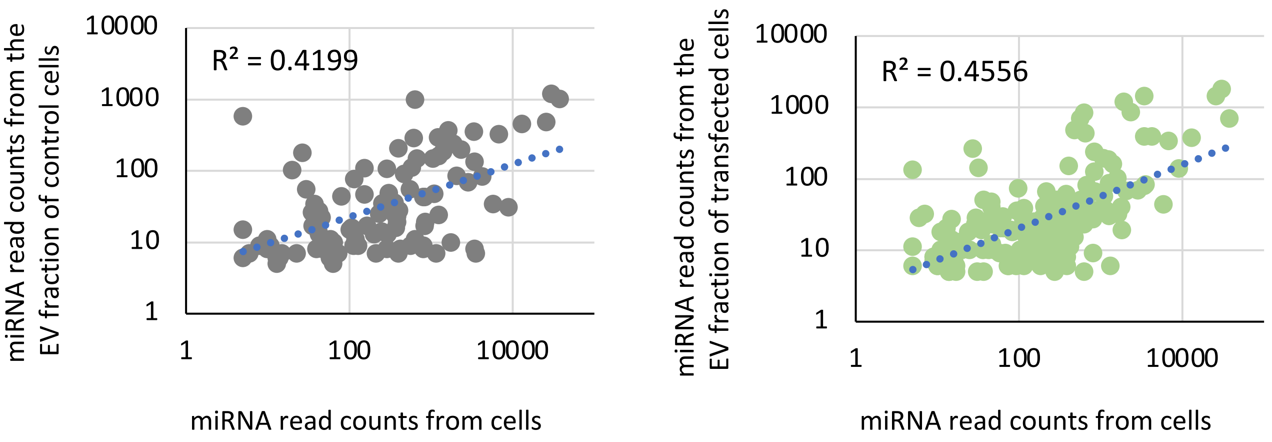

Second experiment

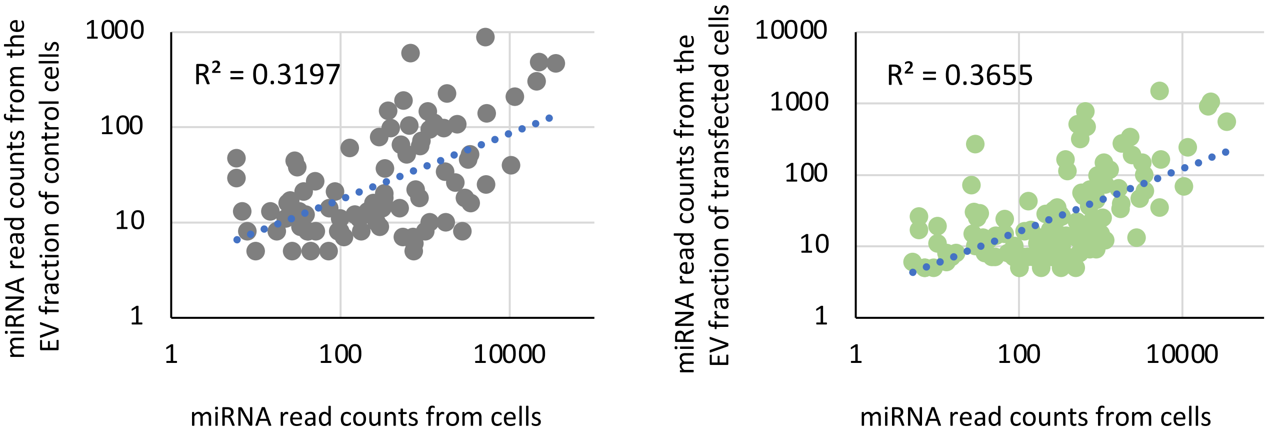

Supplemental Figure 2 Correlation plot of the read counts for the detected miRNAs (with a threshold of ≥ 5 read counts) in the cells and EV fractions obtained from two independent experiments.

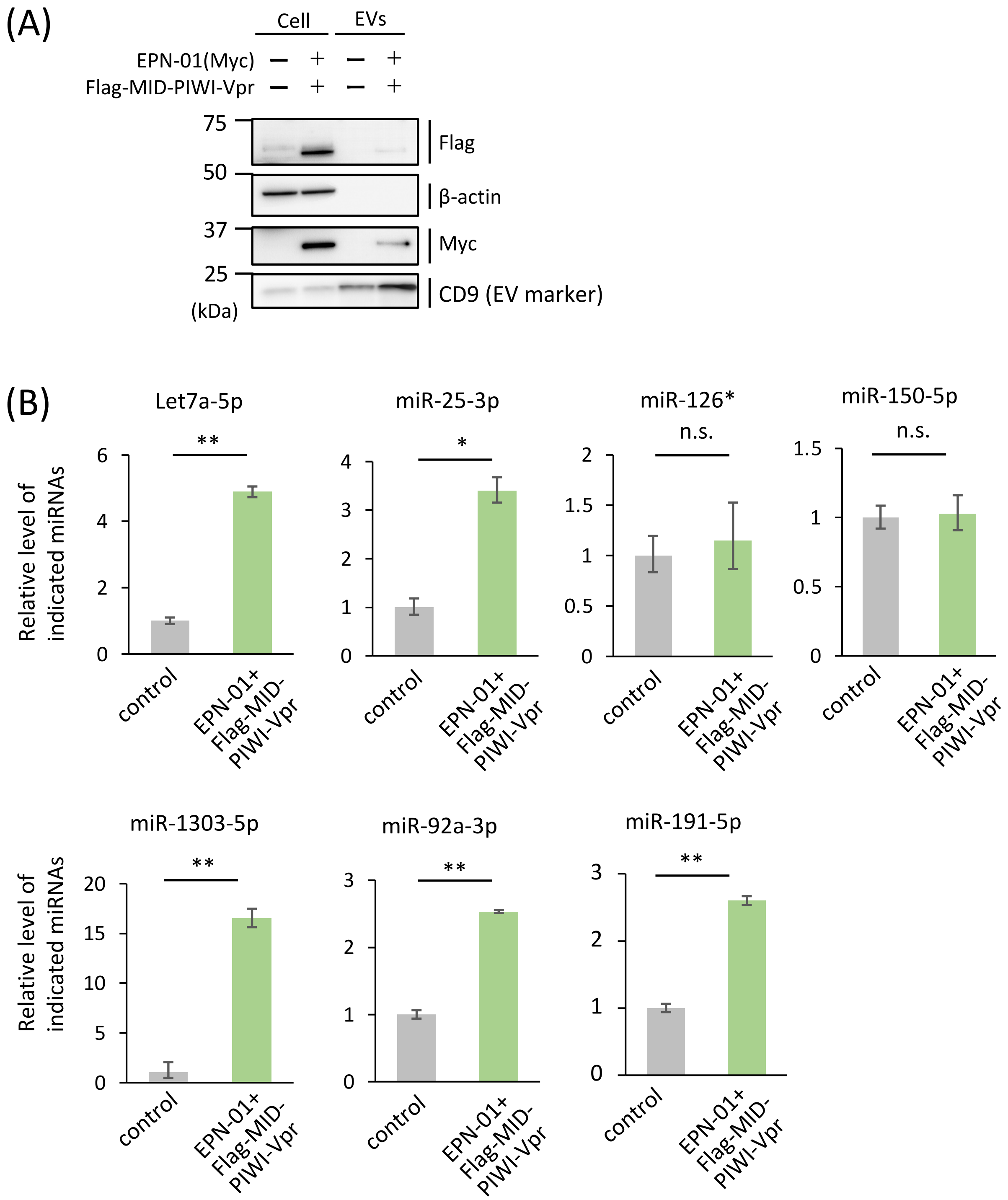

Supplemental Figure 3 (A) Immunoblot showing MID-PIWI fused with Flag-tag and Vpr, and EPN-01 (Myc-tag) harvested from non-transfected or transfected HeLa cells (Cell) or the EV fraction of the cell culture supernatants (EVs). (B) RT-qPCR showing the relative levels of indicated miRNAs from non-transfected (control) or transfected (EPN-01+Flag-MID-PIWI-Vpr) HeLa cells. PCR reactions for each sample were carried out in triplicate. Data shown are $2^{-\Delta Ct}$ value expressed relative to the mean determined for the control, which was normalized to 1; results are representative of three independent experiments. Error bars represent the standard deviation. *p < 0.05, **p < 0.01, Student's t-test was performed using Ct value of three independent experiments.

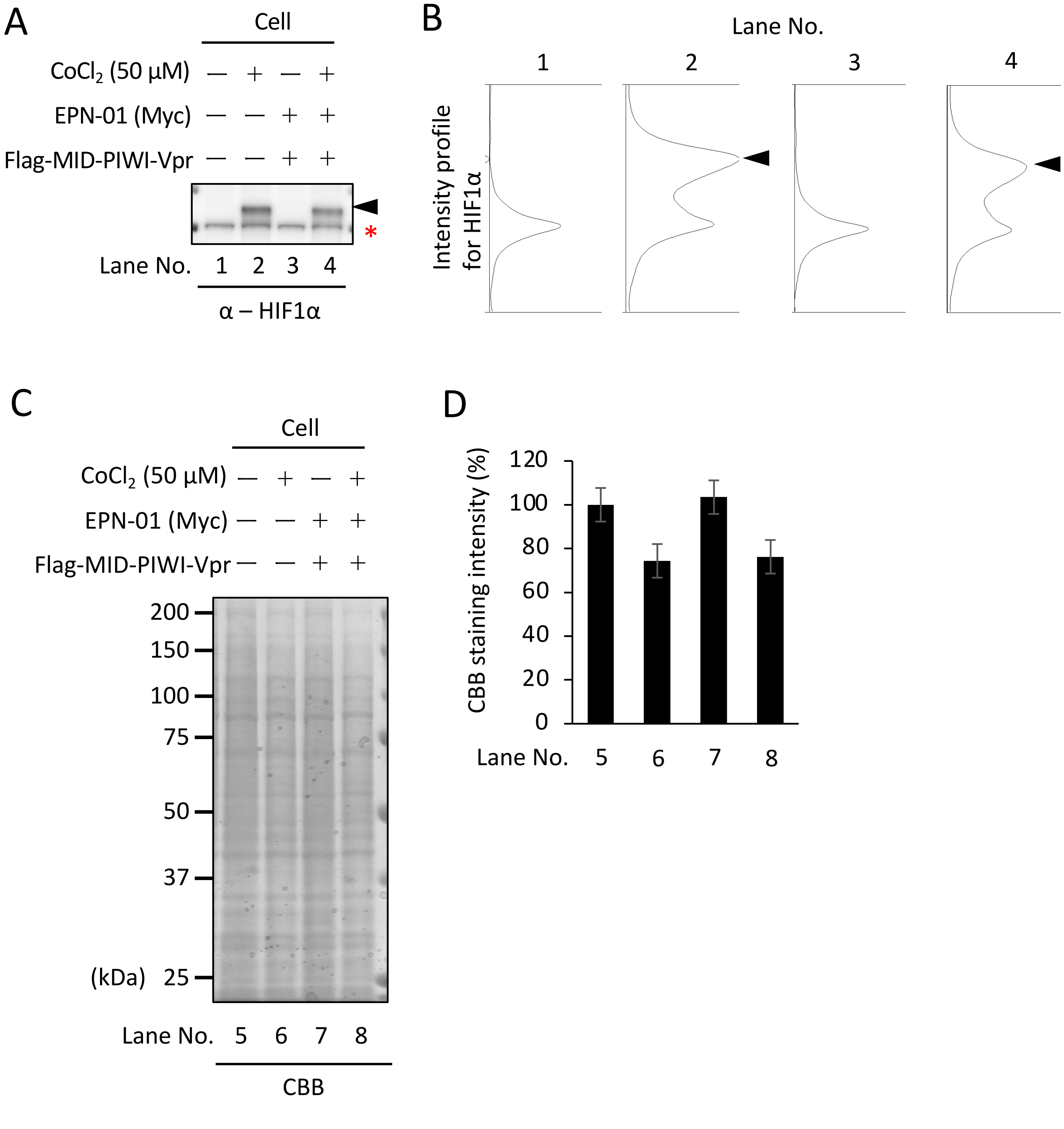

Supplemental Figure 4 (A) Immunoblot showing the accumulation of HIF1α in HEK293T treated with $CoCl_2$ (50 µM) normalized to the total protein measured by BCA protein assay. The black arrowhead indicates HIF1α (red asterisk, non-specific bands). (B) Densitogram of lane 1 – 4 in Fig.S3A. The black arrowhead indicates the peak of the HIF1α band. (C-D) Coomassie Brilliant Blue (CBB) staining of cell lysates used for Fig. 4A (Cell) (C) and CBB staining intensity of lanes 5 – 8 in Fig.S3C (D). Data are the mean ± standard error of three independent experiments.

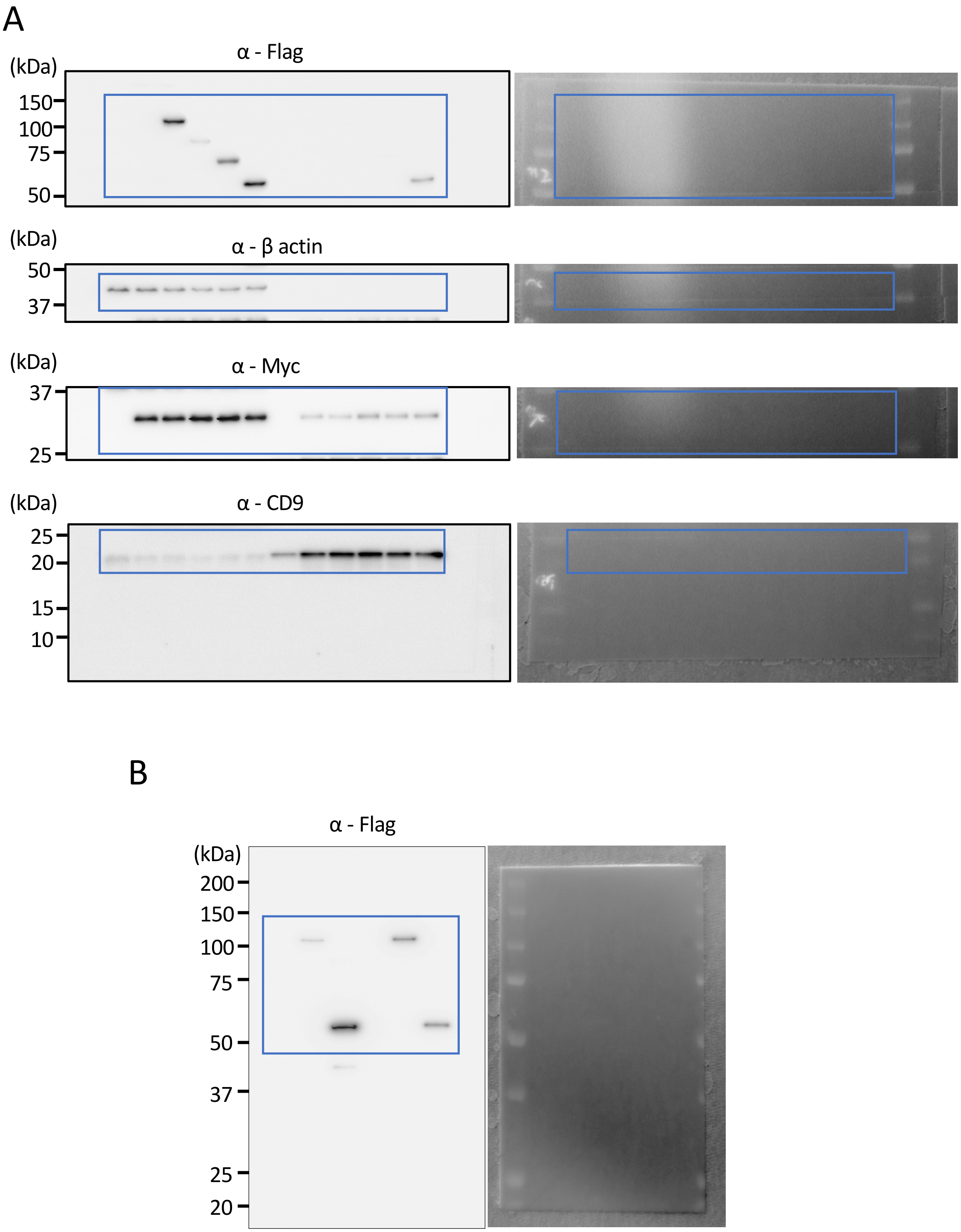

Supplemental Figure 5 Uncropped immunoblot images. (A) Figure 1C, (B) Figure 1D (left: chemiluminescence, right: membrane). Cropped images highlighted by blue boxes.

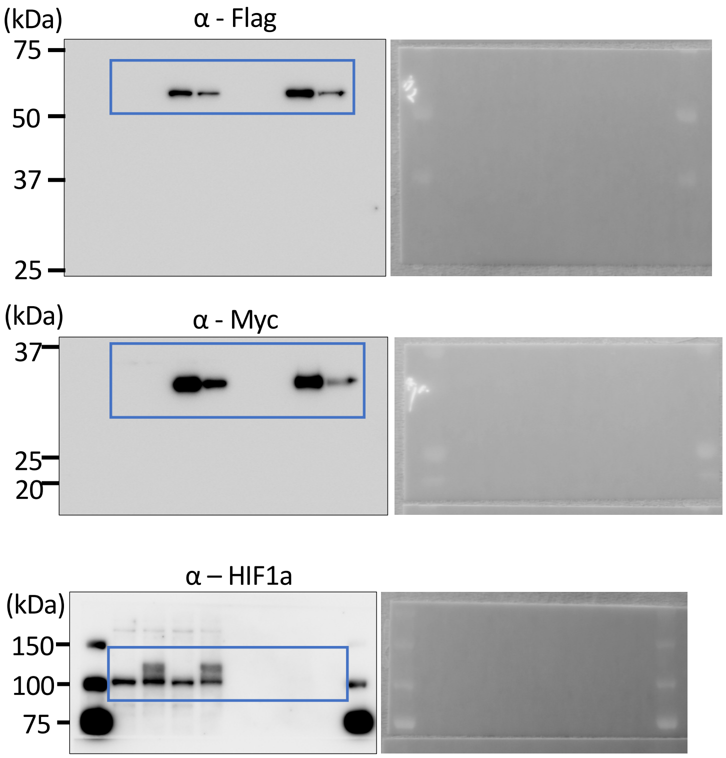

Supplemental Figure 6 Uncropped immunoblot images presented in Figure 4A (left: chemiluminescence, right: membrane). Cropped images highlighted by blue boxes.

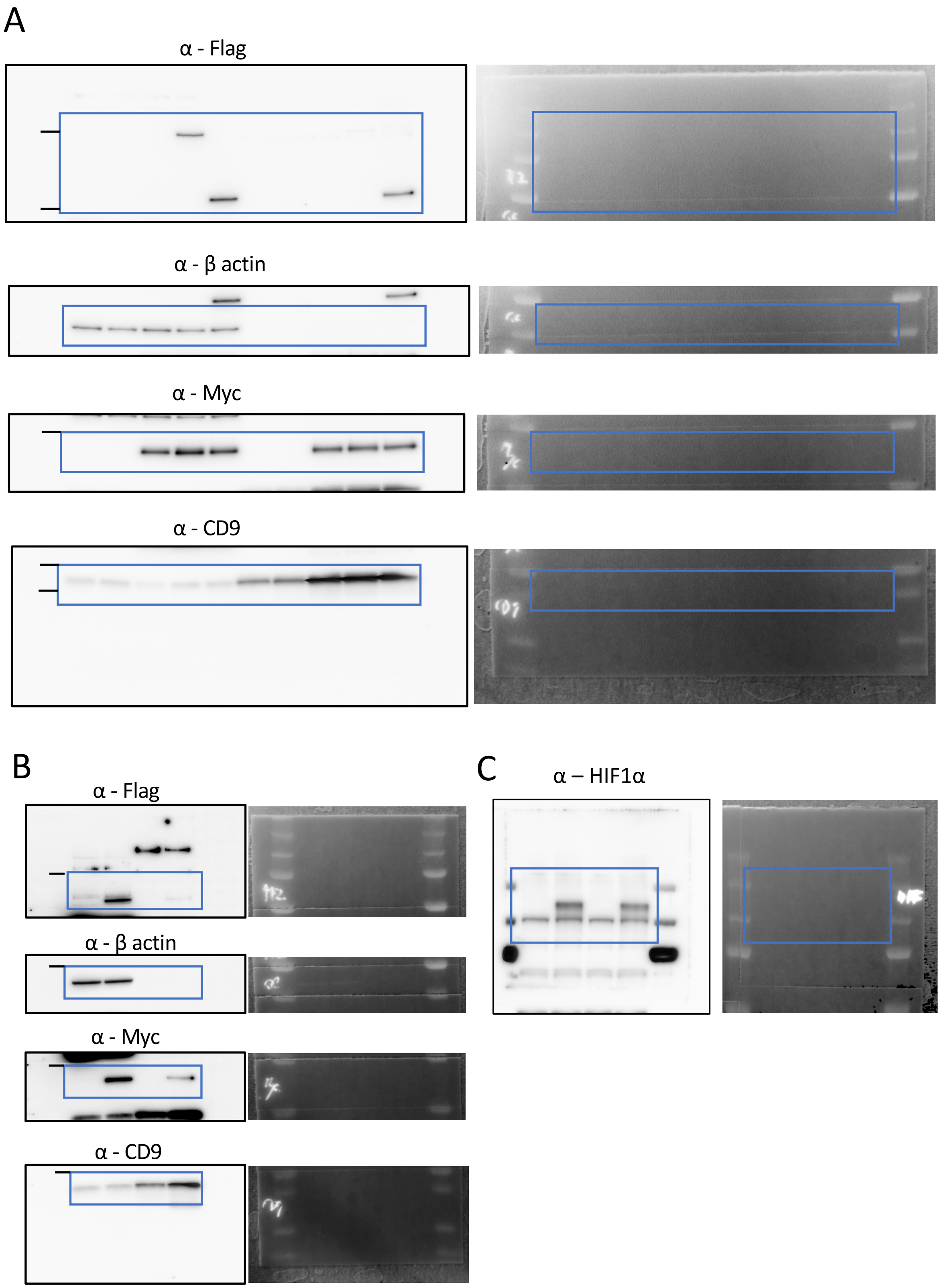

Supplemental Figure 7 Uncropped immunoblot images. (A) supplemental Figure 1A, (B) supplemental Figure 2A, (C) supplemental Figure 3A (left: chemiluminescence, right: membrane). Cropped images highlighted by blue boxes.
